# Directional Field-Dependence of Magnetoimpedance Effect on Integrated YIG/Pt-Stripline System

**DOI:** 10.3390/s21186145

**Published:** 2021-09-13

**Authors:** Arthur L. R. Souza, Matheus Gamino, Armando Ferreira, Alexandre B. de Oliveira, Filipe Vaz, Felipe Bohn, Marcio A. Correa

**Affiliations:** 1 Departamento de Física, Universidade Federal do Rio Grande do Norte, Natal 59078-900, Brazil; arthur_souza77@yahoo.com.br (A.L.R.S.); mgamino@fisica.ufrn.br (M.G.); abo1980@gmail.com (A.B.d.O.); felipebohn@fisica.ufrn.br (F.B.); 2 Centro de Física, Universidade do Minho, 4710-057 Braga, Portugal; armando.f@fisica.uminho.pt (A.F.); fvaz@fisica.uminho.pt (F.V.)

**Keywords:** magnetization dynamics, magnetoimpedance, YIG

## Abstract

We investigated the magnetization dynamics through the magnetoimpedance effect in an integrated YIG/Pt-stripline system in the frequency range of 0.5 up to 2.0 GHz. Specifically, we explore the dependence of the dynamic magnetic behavior on the field orientation by analyzing beyond the traditional longitudinal magnetoimpedance effect of the transverse and perpendicular setups. We disclose here the strong dependence of the effective damping parameter on the field orientation, as well as verification of the very-low damping parameter values for the longitudinal and transverse configurations. We find considerable sensitivity results, bringing to light the facilities to integrate ferrimagnetic insulators in current and future technological applications.

## 1. Introduction

The giant magnetoimpedance (MI) effect corresponds to the strong variation of the electrical impedance of a soft magnetic material when submitted to an external magnetic field [1,2,3,4,5,6,7,8,9]. Since the discovery of the effect, MI has attracted attention due to its versatility. In the context of fundamental physics, the effect is as an exciting alternative, with some potential advantages, to the traditional ferromagnetic resonance (FMR) effect. The fields configuration in the MI experiment is quite similar to that verified in the traditional FMR measurements [10,11]. However, while FMR makes use of a resonant cavity having a fixed frequency, the MI effect, in turn, allows us to explore the evolution of the ferromagnetic resonance with frequency and field strength in both saturated and unsaturated magnetic states. Hence, from the technological point of view, the MI effect arises as a sharp tool to detect small magnetic field changes. In addition, materials exhibiting MI appear as sensitive field sensor elements that can be integrated into a wide variety of electronic devices [11,12,13,14,15,16].

In recent years, the interest in the MI effect has increased, which at first glance may be based on the increasing demand for biosensors [12,17,18,19,20,21]. Within this field, different groups have recently reported interesting results. For instance, Kurlyandskaya and colleagues [17] have explored the MI response of a Co-based alloy ribbon composing a MI-sensitive element. In this case, the studied ribbon is quite thick, favoring the obtainment of high MI variations at the low frequency regime, an essential feature to the integration of the MI-sensor elements into devices. Following a different line, Yu and coworkers [12] have investigated the MI effect in thin films as a sensor to detect magnetic particles in blood vessels. Remarkably, the analyzed multilayer films present significant MI sensitivity despite the smaller thickness of the samples, reaching up to 41%/Oe for low field variations at the moderate frequency regime.

However, in the meantime, the search for electronic devices characterized by low energy consumption and low cost of production has been the main reason for the renewed attention to the MI effect.

Owing to this area, the nanostructured systems engineered to these ends have enabled us to scrutinize numerous spintronics effects. Among the several materials taken into account to the building of the spintronic nanostructures, ferrimagnetic insulators (FMI) as Y${}_{3}$Fe${}_{5}$O${}_{12}$ (YIG) alloy deserves notability, bringing unique features that are advantageous in this context over the ones of other materials, including magnetic conductors [22,23,24,25]. Specifically, the electrical nature, magnetic moment, and very-low damping parameter make YIG the ideal playground for investigations in which pure spin currents are considered, suppressing the charge current [26,27].

The magnetization dynamics in YIG films have been extensively probed through broadband ferromagnetic resonance in recent years; but the link between FMI materials and MI experiments remained elusive until recently [28,29,30,31]. With this spirit, connecting YIG and MI, Kang and coworkers [28], for instance, have addressed the dynamics in YIG spheres and a single-crystalline YIG film deposited by using liquid phase epitaxy. The authors have disclosed very interesting results by employing a vector network analyzer (VNA) to perform MI measurements in a wide range of frequencies, with MI ratio values of around 256% and MI sensitivity of ∼8.8%/Oe for such systems. Next, Madwal and colleagues [29] have investigated a sputtered YIG single-layer thin film with a thickness of 45 nm. In this case, the magnetoimpedance experiments have been performed using the inductive method, in which a signal coil is wound around the film and surprisingly, despite the reduced dimension of the film, the FMR effect is found even at the low-frequency regime, from 0.5 up to 2.0 GHz.

Remarkably, the studies aforementioned deal with techniques in which the sample is electrically disconnected from the measurement system, which complicates the integration of the samples as MI-sensor elements in an electronic device. It is worth remarking that part of the difficulty in obtaining MI results in YIG samples resides in the high resistivity of the material. Nevertheless, recently, it has been shown that the growth of YIG/NM heterostructures, where NM is a non-ferromagnetic metal such as Ag, Cu, and W [30,31], may act as a way to circumvent this adversity and perform investigations on magnetization dynamics through the MI effect in the low frequency regime. In these previous works, the YIG/NM heterostructures were entirely produced by using the Magnetron Sputtering technique. This experimental procedure limits the reach of YIG with high thicknesses. Moreover, the NM materials in these previous studies present low spin–orbit coupling when compared with Pt material. Therefore, the modification of the YIG deposition technique (allowing for increased thickness) and the use of Pt material can bring interesting results, mainly in the MI response at moderate- and high-frequency regime.

For these heterostructures, beyond obtaining from the MI measurements important magnetic parameters such as effective magnetization $M_{eff}$ and effective damping parameters $\alpha_{eff}$, fingerprints of the FMR effect have been identified. Consequently, it has been demonstrated that these YIG/NM-stripline systems are promising candidates as MI-sensor elements to integrate magnetic devices.

This article reports a systematic investigation of the magnetization dynamics through the MI effect in an integrated YIG/Pt-stripline system. Specifically, we explored the dependence of dynamic magnetic behavior on field orientation. We went beyond the traditional longitudinal MI (LMI) effect, in which magnetic field and probe current are parallel, and also acquired the MI response for the transverse (TMI) and perpendicular (PMI) setups. From the results, we disclose the strong dependence of the effective damping parameter on the field orientation, as well as verify very-low damping parameter values for the LMI and TMI configurations. The observed high MI sensitivity and the experimental setup employed here turn easy to integrate ferrimagnetic insulators in current and future technological applications.

## 2. Experiment

To engineer the integrated YIG/Pt-stripline system, we considered a Y${}_{3}$Fe${}_{5}$O${}_{12}$/Pt bilayer grown in two steps. First, we produced a YIG film with a thickness of 6μm by Liquid Phase Epitaxy (LPE) onto a (111) Gadolinium Gallium Garnet (GGG) substrate with dimensions of 5×5 mm. After, we covered the YIG with a 6-nm-thick Pt layer deposited by magnetron sputtering. The deposition process was carried out using a Pt target (99.99% of purity) with the following parameters: base pressure of $5\times 10^{-8}$ Torr, deposition pressure of $3\times 10^{-3}$ Torr, with 99.99% pure Ar at 20 sccm constant flow, and using a DC source with a current of 50 mA.

The structural features of the sample were verified through X-ray diffraction (XRD). The XRD experiment was performed in the θ−2θ geometry, with Cu-$K_{\alpha }$ radiation (λ=1.54060 Å), using a Rigaku Miniflex II system.

The quasi-static magnetic behavior was obtained through magnetization curves at room temperature, acquired using a Vibrating Sample Magnetometer (VSM) Lakeshore model 7407. In particular, the magnetization curves were taken with the magnetic field applied along distinct orientations, corresponding to the very same field configurations employed to the MI experiments.

**Figure 1:**
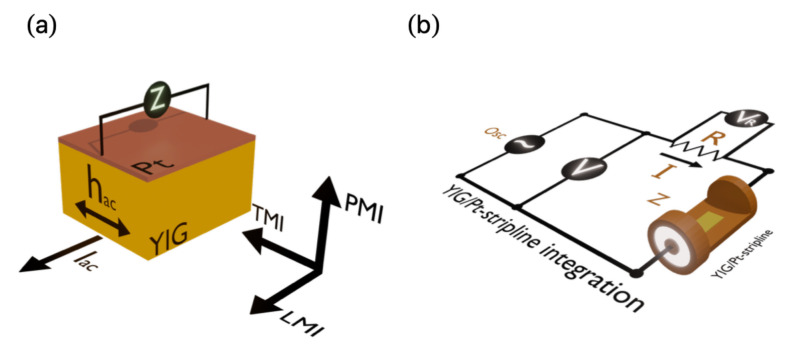
Schematic representation of the MI experiments. (**a**) LMI, TMI, and PMI setups employed in the dynamic magnetic characterization. (**b**) YIG/Pt-stripline system integrated in a circuit as a MI-sensor element.

The MI measurements were performed using a RF-impedance analyzer Agilent model E4991, with an E4991A test head connected to the integrated YIG/Pt-stripline system, in which the bilayer is the central conductor, and it is separated from the ground plane by the substrate. The electrical contacts between the YIG/Pt bilayer and the stripline system were made with 24 h cured low-resistive silver paint. To avoid propagative effects and acquire just the sample contribution to MI, the RF impedance analyzer was calibrated at the end of the connection cable by performing open, short, and load (50Ω) measurements using reference standards. The probe current is fed directly to one side of the sample, while the other side is in a short circuit with the ground plane. We went beyond the traditional longitudinal MI (LMI) effect, in which external magnetic field and probe current are parallel, and also acquired the MI response for the transverse (TMI) and perpendicular (PMI) setups. Specifically, in the TMI configuration, the external magnetic field and probe current are transverse, and the field remains in the plane of the film; in the PMI one, in turn, the field is perpendicular to the film plane. A schematic representation of the MI setups explored here is depicted in Figure 1a. For the LMI case, we employed a solenoid as a source for the magnetic field, with a maximum amplitude of ±300 Oe, while for the TMI and PMI cases, we used an electromagnet, thus reaching ±1500 Oe. While the external magnetic field was varied for all experiments, a 0 dBm (1 mW) constant power was applied to the sample, characterizing a linear regime of driving signal. MI measurements were taken over a frequency range between 0.5 and 2.0 GHz. The frequency sweep was made for each field value, and the real *R* and imaginary *X* components of the impedance *Z* are simultaneously acquired. In the meantime, in our RF-impedance analyzer, the test head may estimate the *Z* from the ratio between the electrical voltage and current, both acquired as sketched in Figure 1b. Then, its maximum amplitude for a given field strength and frequency is obtained by

(1) $$Z=\frac{V}{I}=\frac{V}{V_{R}}R.$$

where *V* is the peak voltage provided by the test head, $V_{R}$ is the potential difference between the terminals of a reference resistor *R*, and *I* is the peak current flowing through the sample. In this sense, such a configuration allows us to wonder a simple circuit with an MI-sensor element to be embarked in a magnetic device. In order to make easier a direct comparison between the measurements, we show here discounted values of the impedance, given by

(2) $$\Delta Z=Z\left(H\right)-Z\left(H_{max}\right),$$

where Z(H) is the electrical impedance for a given external magnetic field value and $Z(H_{max})$ is the impedance value for the maximum external magnetic field, where the sample is saturated magnetically. It is worth pointing out that similar definitions of variation were also taken for the real *R* and imaginary *X* components of the impedance, i.e.,

(3) $$\Delta R=R\left(H\right)-R\left(H_{max}\right)$$

and

(4) $$\Delta X=X\left(H\right)-X\left(H_{max}\right).$$

## 3. Results and Discussion

**Figure 2:**
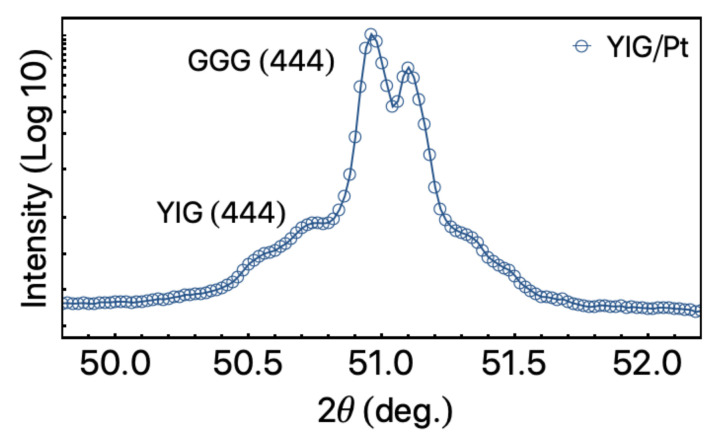
X-ray diffraction result for the YIG/Pt bylayer grown onto a (111) GGG substrate. The peaks are indexed considering the ICSD cards 9237 and 80139 for the GGG and YIG, respectively.

Figure 2 shows the XRD result for our YIG/Pt bilayer. The diffractogram reveals two peaks at $2\theta \approx 51.1^{\circ }$, which are associated with the GGG (111) substrate (ICSD 9237) and ascribed to the coexistence of the $K_{\alpha 1}$ and $K_{\alpha 2}$ contributions. In addition, a low-intensity peak is found at $2\theta =50.9^{\circ }$, assigning the YIG (444) preferential growth (ICSD 80139). The peaks of YIG (444) and GGG (444) overlap due to their good lattice match, and no evidence of polycrystalline YIG is verified, meaning that textured YIG layer is formed in the growth direction of [111]. Our findings are in accordance with results previously reported in the literature for similar heterostructures [32,33,34,35].

**Figure 3:**
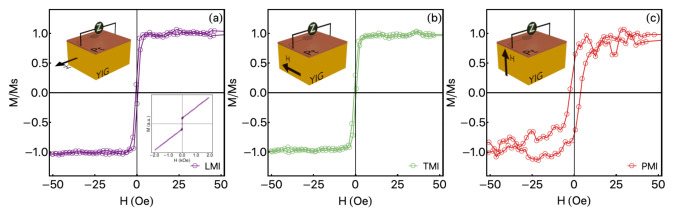
Normalized magnetization curves for the YIG/Pt bilayer acquired with the field along different orientations, (**a**) LMI, in which the external magnetic field is parallel to the drive current on the MI experiment. (**b**) TMI, here the external magnetic field is applied in the film-plane, and perpendicular to the drive current (MI experiment). (**c**) PMI configurations, which the external magnetic field is perpendicular to the film-plane. It is worth mentioning that the paramagnetic contribution of the GGG substrate is removed from each curve and, therefore, only the ferrimagnetic response of the YIG is seen here. The inset in (**a**) shows a representative example of the magnetization curves before the remotion of the paramagnetic contribution.

Although the XRD pattern shows evidence of the YIG crystallization, which is also corroborated from the comparison with the literature, we yet may employ quasi-static magnetic technique to further characterize the YIG phase after the annealing. Figure 3 shows the normalized magnetization curves acquired with the field along different orientations, LMI, TMI, and PMI (see Figure 1a). Notice that only the ferrimagnetic response of the YIG is seen here, given the paramagnetic contribution of the GGG substrate is removed from each curve. Remarkably, the YIG/Pt bilayer has quite weak anisotropic in-plane magnetic properties [36], depicted by the similar magnetization curves acquired for the LMI and TMI configurations. The results suggest soft magnetic properties, with low saturation field $H_{s}$, low coercive field $H_{c}$, and high magnetic permeability. Specifically, we find $H_{s}$ values of 4.2 Oe and 4.5 Oe, while $H_{c}$ ones of 0.5 Oe, from the LMI and TMI experiments, respectively. On the other hand, for the PMI one, the shape of the curve is completely modified. The change is attributed to the shape magnetic anisotropy, which leads to a significant increase of the coercive and saturation fields, reaching to $H_{c}\approx 3.0$ Oe and $H_{s}\approx 14$ Oe. In addition, it is worth highlighting that we observe a drastic reduction of the effective magnetization in the PMI measurement, a fact not identified in the plot since the curve is normalized, but that can be inferred here from the decrease in the signal-to-noise ratio.

The ferrimagnetic behavior and the coercive field value verified here from the magnetization curves, when combined with the XRD result, are indicators of the quite-good quality of our YIG/Pt heterostructure. However, the general features of the whole film, including its magnetic and electrical properties, are essential issues for the MI response. As a consequence, in order to make it feasible to carry out MI experiments in magnetic insulators and place them as MI-sensor elements for magnetic devices, we overcome any experimental adversity due to the high electrical resistivity of the material by capping the YIG with a non-magnetic conductor Pt layer.

It is well known that the magnetic properties of our YIG/Pt bilayer are reflected in the magnetization dynamics, including the magnetoimpedance effect [2,10]. These features establish the limits in which distinct mechanisms command the MI response. Here, the soft magnetic behavior and the integration between the YIG/Pt bilayer and the stripline system allow us to observe FMR contributions even in the low-frequency regime. Consequently, we can induce substantial MI modifications, making the integrated system a promising candidate for sensor elements.

**Figure 4:**
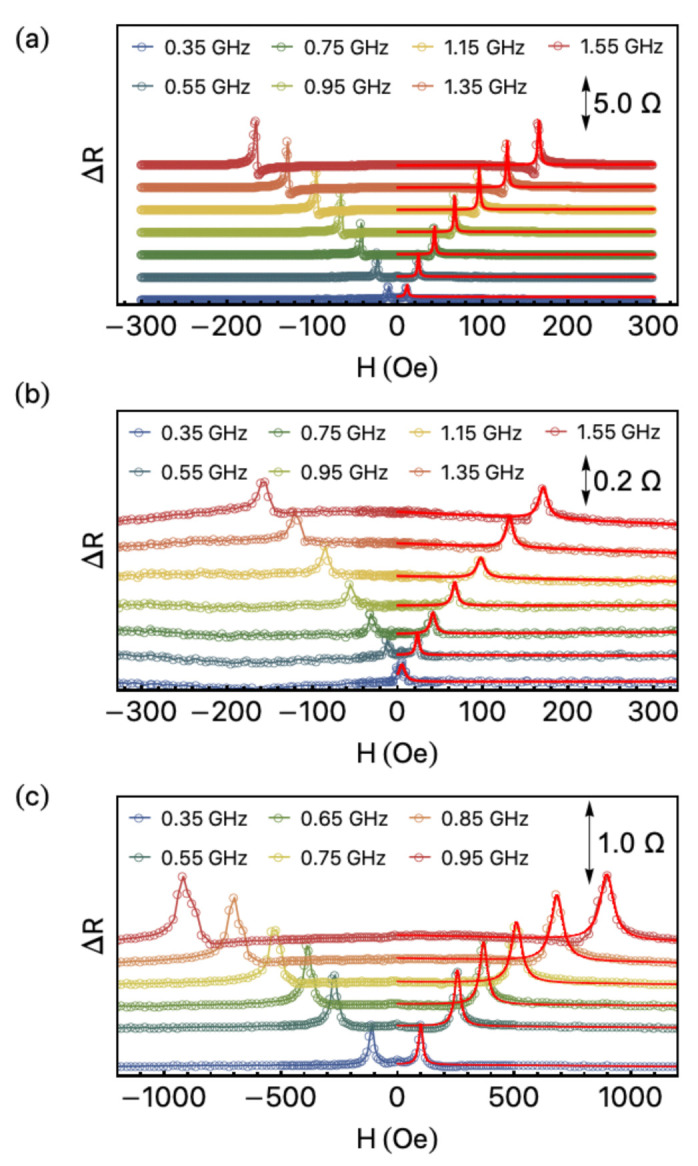
Variation of the real component of the impedance, ΔR, as a function of the external magnetic field for selected frequency values. Response of the YIG/Pt-stripline system for the (**a**) LMI, (**b**) TMI, and (**c**) PMI configurations. The curves are shifted on a vertical scale in order to make the visualization clearer. The symbols correspond to the experimental data, while the red lines are the fit from which the resonance field $H_{r}$ and linewidth ΔH are obtained. The arrows indicate the scales of each measured configuration with the appropriate values.

Figure 4 shows the evolution of ΔR as a function of the external magnetic field with the frequency. The curves were acquired over a complete magnetization loop and present hysteretic behavior. However, here we show just part of the curves, when the field goes from the negative to maximum positive value.

At first glance, we notice the curves have similar general shapes. Nevertheless, a closer inspection reveals that the ones acquired for the PMI configuration occur in a field range dissimilar to the one verified for the LMI and TMI ones. This feature is due to the substantial modification in the shape anisotropy and, consequently, in the anisotropy field. In addition, for all experiments, we observe the amplitude of the peaks is dependent on the field orientation. More specifically, the peak amplitude and the peak’s position in field are a result of the orientation between field and magnetization, and of the interplay of the effects associated with the magnetic anisotropy field and damping parameter [37].

From the general point of view, our samples have all classical features of the magnetoimpedance observed in conducting ferromagnetic systems. Specifically, the curves exhibit a double peak behavior, symmetrical around H=0, for the whole frequency range, irrespectively of the field configuration. An interesting feature related to the ΔR behavior resides in the dependence of the position peaks with probe current frequency. We observe the displacement of the peaks towards higher fields as the frequency increases, even for the smallest frequency values. Such peak behavior is a fingerprint of the FMR effect controlling the magnetization dynamics and the MI variations, in a response similar to that obtained through the broadband FMR technique.

From the ΔR results, the resonance field $H_{r}$ and the linewidth ΔH were estimated by fitting the peaks using a Lorentzian function, as shown in Figure 4. Such quantities are key parameters for obtaining the effective magnetization $M_{eff}$ and the effective damping parameter $\alpha_{eff}$. Specifically, the dependence of $f_{r}$ with $H_{r}$ provides $M_{eff}$ through the Kittel equation

(5) $$f_{r}=\frac{\gamma }{2\pi }\sqrt{\left(H_{r}+H_{k}\right)\left(H_{r}+H_{k}+4\pi M_{eff}\right)},$$

in which γ/2π is the gyromagnetic ratio and $H_{k}$ is the anisotropy field. In addition, $\alpha_{eff}$ is achieved from the relation between ΔH and $f_{r}$,

(6) $$\Delta H=\Delta H_{\circ }+\frac{2\pi f\alpha_{eff}}{\gamma },$$

where $\Delta H_{\circ }$ is the extrinsic inhomogeneous contribution to ΔH and *f* is the frequency, i.e., $f_{r}$.

**Figure 5:**
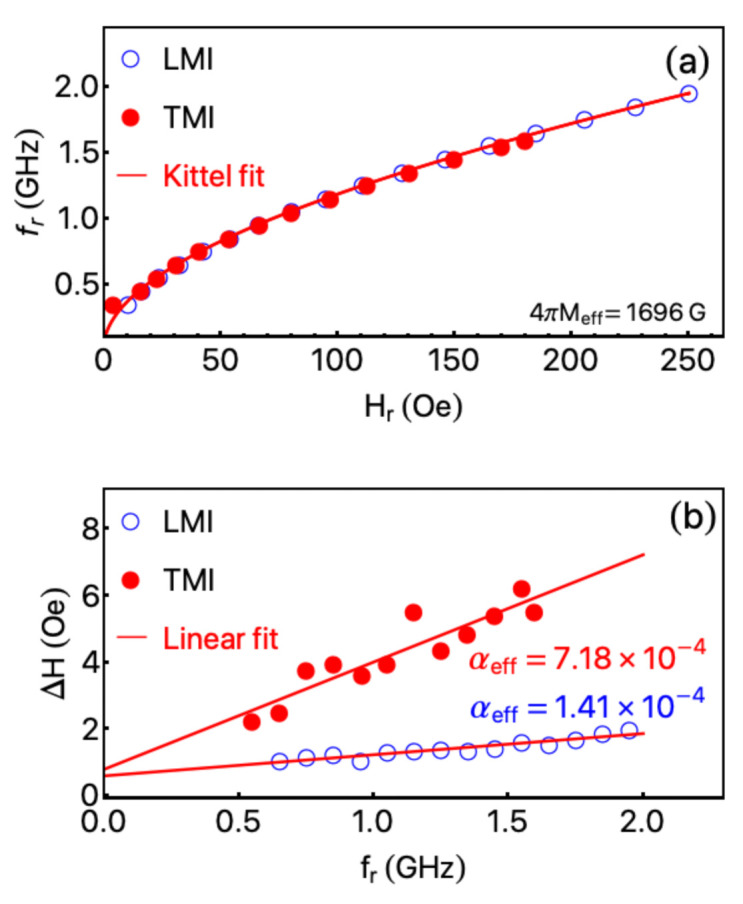
(**a**) Resonance frequency $f_{r}$ as a function of resonance field $H_{r}$ of the YIG/Pt-stripline system for the LMI and TMI configurations. The symbols are the experimental data and the red lines correspond to the fit obtained with the Kittel equation, Equation (5), then inferring the effective magnetization $M_{eff}$. (**b**) FMR linewidth ΔH as a function of the $f_{r}$ in the LMI and TMI configurations. The symbols are the experimental data and the red lines correspond to the possible fit obtained with Equation (6), then providing an estimate of the effective damping parameters $\alpha_{eff}$.

Figure 5 brings both plots, $f_{r}$ vs. $H_{r}$ and ΔH vs. $f_{r}$. From Figure 5a, we verify the dependences of $f_{r}$ with $H_{r}$ for the LMI and TMI configurations are similar, as expected due to the quite-weak anisotropic in-plane magnetic properties. Assuming γ/2π=2.8 GHz/kOe and $H_{k}=0.5$ Oe, this latter close to the $H_{c}$ values obtained from the quasi-static magnetization curves, we infer $4\pi M_{eff}\approx 1696$ G using Equation (5). The $4\pi M_{eff}$ value is in concordance with results previously reported in the literature [38]. For the PMI configuration, not shown here, the effective magnetization is significantly smaller, which is attributed to the shape anisotropy due to the reduced thickness.

From Figure 5b, we identify the relations between ΔH and $f_{r}$ for the LMI, TMI. Generally, our system consists of a ferrimagnetic insulator capped by a non-magnetic metallic layer. Given Pt is a metal with high spin–orbit coupling, the effective damping parameter in our sample has contributions of distinct mechanisms. The first one is the well-known Gilbert damping parameter. Such contribution consists of representing the relaxation mechanisms by a torque that pulls the magnetization toward the equilibrium direction [39]. Moreover, considering that the longitudinal and transverse components of the magnetization are stirred through different relaxation rates, the Bloch–Bloembergen phenomenology [39] is present in our sample. The second contribution to the effective damping parameter comes from the two-magnon and spin pumping mechanisms, especially due to the bilayer structure of our sample. There are numerous interesting studies playing with the mechanisms influencing $\alpha_{eff}$ and bringing the theoretical background to understand contributions for a given system [39,40,41,42,43]. Here, we do not address such issue in detail and instead focus our efforts in the fit using Equation (6) to infer the effective damping parameters and the extrinsic inhomogeneous contribution to ΔH. Once the effective damping ($\alpha_{eff}$) is the parameter considered for the future sensor applications.

For the LMI and TMI configurations, we find $\alpha_{eff}$ of $1.41\times 10^{-4}$ and $7.18\times 10^{-4}$, respectively. Remarkably, such values are very low and are in agreement with results for YIG films [43,44]. Moreover, the in-plane inhomogeneous contribution to ΔH seems to be the same for both, as expected. It is well known $\alpha_{eff}$ has a central role in the magnetic response of the sample. For instance, samples with low a $\alpha_{eff}$ reach the magnetic stabilization quickly, an important parameter for a sensor in which the fast magnetic response is primordial, as is the case of biosensors. For the PMI one, in turn, we are not able to fit the $\alpha_{eff}$ due to the mechanisms associated with the non-uniform excitation modes and domain contribution what leads to a considerable increase in the ΔH as observed in Figure 4c. Nevertheless, the $\alpha_{eff}$ value for the PMI setup seems to be comparable with those found for other interesting ferromagnetic systems, such as thin films of Co_2_FeAl full-Heusler alloy [45,46].

**Figure 6:**
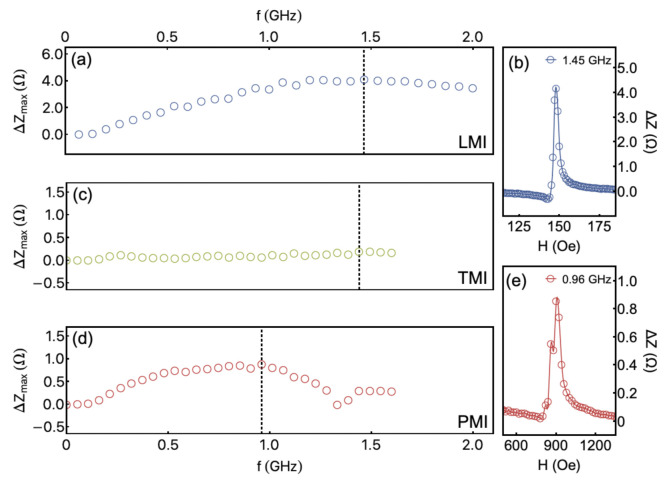
(**a**) Maximum ΔZ value, $\Delta Z_{max}$ as a function of the frequency for the LMI configuration. The maximum efficiency is found for f=1.45 GHz, as indicated by the dashed line. (**b**) The ΔZ vs. *H* at f=1.45 GHz, as a representative example of the analyzed curves. The $\Delta Z_{max}$ is achieved from the difference between the *Z* value at the peak and the *Z* at the maximum magnetic field. (**c**) $\Delta Z_{max}$ as a function of the frequency for the TMI configuration, whose maximum at 1.4 GHz is indicated by the dashed line. (**d**) A similar plot for the PMI configuration. (**e**) The ΔZ vs. *H* at 0.96 GHz for the PMI setup.

With the straight potential for sensor applications, we focus on the MI performance as a function of the frequency and field strength. Figure 6 shows the maximum ΔZ value, $\Delta Z_{max}$, as a function of the frequency for the LMI, TMI, and PMI configurations. Such analysis allows us to infer the frequency range in which the sensor element has the best MI efficiency. For LMI, the highest $\Delta Z_{max}$ takes place at around 1.45 GHz and is found for an external magnetic field of 150 Oe, as can see in Figure 6a,b. We observe a monotonic rise of $\Delta Z_{max}$ up to frequencies close to 1.0 GHz, where the curve reaches a constant value within the experimental error. This behavior is interesting since the frequency may be modified without losing the MI efficiency of the sensor. The $\Delta Z_{max}$ behavior for the TMI configuration is quite similar to that discussed for the LMI one. However, as we can confirm from Figure 6c, the MI efficiency is very low, becoming negligible if compared to the results acquired for the other experimental setups. Although we observe low $\alpha_{eff}$ value for this field configuration, the alignment between the external magnetic field, alternating magnetic field, and magnetization seem to affect the MI variations drastically, vanishing $\Delta Z_{max}$. At last, Figure 6d,e shows the results for the PMI configuration. In this case, the MI efficiency presents an initial increase up to 0.5 GHz, achieving a roughly constant value between 0.5 and 1.0 GHz, followed by a decrease above 1.0 GHz. Then, the highest $\Delta Z_{max}$ is found at around 0.96 GHz. It is worth highlighting such result is found at 900 Oe, in a sense the PMI configuration allows the identification of interesting MI efficiency values at the high-field regime.

These findings bring to light an exciting way to promote the integration of insulating ferrimagnetic materials in sensor elements, and modify the field range in which the optimal MI response is achieved, i.e., simply by changing the orientation of the magnetic field in the experiment.

**Figure 7:**
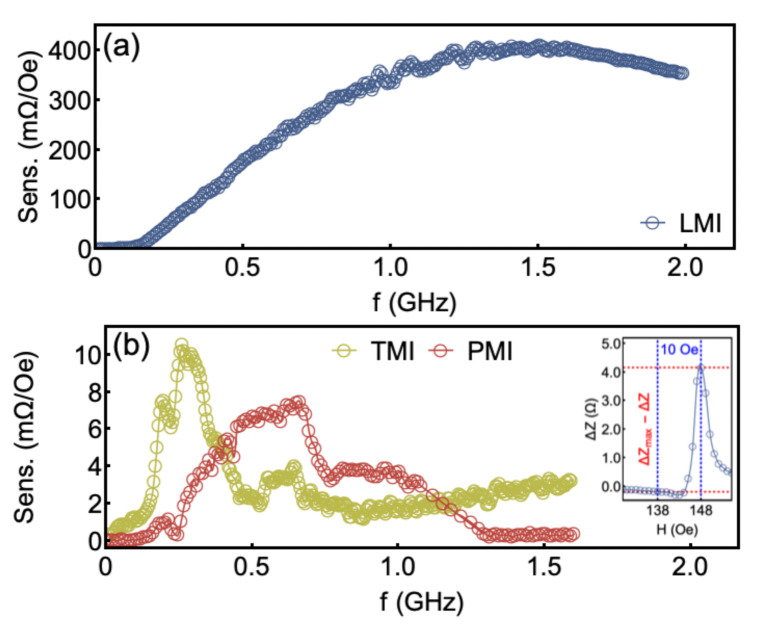
(**a**) Sensitivity as a function of frequency for the LMI field configuration. The maximum value is 415 mΩ/Oe at 1.5 GHz. (**b**) A similar plot for the TMI and PMI configurations. The inset depicts a representative example of the experimental procedure employed to calculate the sensitivity.

The $\alpha_{eff}$ values verified for our integrated YIG/Pt-stripline reveal fingerprints of a low-damping dynamical system. Within this context, the narrow ΔZ peaks as a function of the field provides insights on the MI sensitivity. To quantify the sensitivity as a function of the frequency, we calculate the magnitude of the impedance change

(7) $$\mathrm{Sens}.=\frac{\Delta Z_{max}\left(H\right)-\Delta Z\left(H-10\right)}{10},$$

where $\Delta Z_{max}\left(H\right)$ corresponds to the maximum values of the ΔZ that is observed at the field *H*, and ΔZ(H−10) is the ΔZ observed at H−10 (see inset in Figure 7b).

Figure 7 shows the sensitivity as a function of frequency for the LMI, TMI, and PMI configurations. For LMI, Figure 7a, we observe the maximum value reaches ∼415 mΩ/Oe at 1.5 GHz. However, we point out there is a broad range of frequencies, between 0.5 and 2.0 GHz, in which the sensitivity has significant values, in a sense, we may vary the frequency without loss of sensitivity. For the TMI configuration, we find a narrow range of frequencies with a sensitivity of 10 mΩ/Oe. In particular, such behavior becomes interesting since high sensitivity values are found in a range of 0.2 GHz, taking place at the low frequency regime, below 0.35 GHz. At last, for the PMI configuration, we verify a maximum sensitivity of 7.5 mΩ/Oe at around 0.65 GHz. In this case, it is worth emphasizing such value takes place at high-field values, as previously mentioned (see Figure 6e).

## 4. Conclusions

In summary, we have investigated herein the magnetization dynamics through the magnetoimpedance effect in an integrated YIG/Pt-stripline system. Specifically, we have explored the dependence of dynamic magnetic behavior on field orientation. To this end, we have analyzed the magnetoimpedance response for the traditional longitudinal configuration, as well as for the transverse and perpendicular ones. From the experimental results, we have estimated magnetic parameters that are fundamental for a sensor element, such as the effective magnetization and the effective damping parameter. We have observed low $\alpha_{eff}$ values of $1.41\times 10^{-4}$ and $7.18\times 10^{-4}$ for the LMI and TMI configurations, respectively. Moreover, we have found a significant increase of $\alpha_{eff}$ for the PMI configuration, as expected. From the technological perspective, we have obtained the efficiency $\Delta Z_{max}$ as a function of the frequency and have estimated the sensitivity for our system. Within this context, the change of field configuration suggests the integrated YIG/Pt-stripline system may be used in different magnetic devices as a sensor element. In particular, the LMI configuration reveals high sensitivity for a wide frequency range, in which a change of frequency does not yield any loss of sensitivity, while the TMI and PMI ones disclose high sensitivity in a limited frequency interval. However, for the PMI field configuration, the higher sensitivity happens at high fields, bringing the possibility to explore distinct field ranges with a single sensor. The verified high MI sensitivity brings to light the facilities to integrate ferrimagnetic insulators in current and future technological applications, as ultra-fast sensors.
